# Quantifying vocabulary learning belief and strategy - A validation study of the Vietnamese version of Gu's (2018) vocabulary learning questionnaire

**DOI:** 10.1016/j.heliyon.2023.e16009

**Published:** 2023-05-01

**Authors:** Nguyen Huynh Trang, Duy Vinh Truong, Hung Tan Ha

**Affiliations:** School of Foreign Languages, University of Economics Ho Chi Minh City, Viet Nam

**Keywords:** Vocabulary learning beliefs, Vocabulary learning strategies, Exploratory factor analyses, Confirmatory factor analyses, Structural equation modeling

## Abstract

**Background:**

The field of language teaching and learning has long recognized the role of vocabulary knowledge in all aspects of language proficiency and indicated that vocabulary beliefs and learning strategies play a pivotal role in learners' vocabulary development. As a result, understanding learners’ beliefs and strategies in vocabulary learning is of paramount importance to language teachers. The Vocabulary Learning Questionnaire (VLQ) developed by Peter Gu in 2018 could be considered the most recent, validated instrument for the measurement of vocabulary learning beliefs and strategies. However, the VLQ contains too many items and is only available in English. The objectives of the study, therefore, are (1) to develop and validate a Vietnamese version of the VLQ which can exclude construct-irrelevant noises related to L2 comprehension, and (2) to reduce the number of items while retaining the key factors in the instruments.

**Methods:**

722 Vietnamese university students took part in the study. Exploratory factor analyses (EFA) and confirmatory factor analyses (CFA) were examined with the free software Jamovi 2.3.13. Both Cronbach's alpha and McDonald's omega were employed to evaluate the factors' internal consistency.

**Results:**

Separate EFAs confirmed the two dimensions of vocabulary beliefs, explaining 62.6% of the total variance, and seven factors of vocabulary strategies, predicting 72.1% of the total variance. CFAs confirmed the hypothesized 9-dimensional structures of different vocabulary learning beliefs and strategies and offer cross-validation evidence for the Vietnamese VLQ. Reliability metrics demonstrated acceptable internal reliability for vocabulary belief and strategy sub-scales.

**Conclusion:**

The Vietnamese VLQ provides a validated measure of vocabulary beliefs and strategies. The 30-item version of the Vietnamese VLQ serves as a starting point for future research in the field of vocabulary learning and teaching in Vietnam.

## 1 Introduction

Vocabulary knowledge is arguably the most important factor determining how well people comprehend texts [1]. For decades, psychological research on the relationship between vocabulary knowledge and comprehension has proven the undeniably strong link between the two variables [[2], [3], [4], [5]]. Another interesting way to look at the impact of vocabulary on comprehension is by trying to answer the tough question: “how many words do we need to understand …“. Building on the knowledge that readers or listeners need to be familiar with at least 95% of the words in a text, talk, or recoding to comprehend it adequately [[6], [7], [8], [9], [10]] lexical profiling studies have provided a comprehensive list of lexical demands, or the amount of vocabulary needed to understand 95% of the words in a particular text [[11], [12], [13], [14], [15], [16], [17], [18], [19]].

Realizing the importance of vocabulary knowledge and the active role of learners in their vocabulary development [1,20], scholars soon paid attention to how learners learn vocabulary and the forces that drive learners' vocabulary learning [1,[21], [22], [23], [24]]. One of the most influential questionnaires created to examine learners' beliefs and strategies in vocabulary learning was Gu and Johnson's mega questionnaire which contained 108 questions [22]. The questionnaire was designed for university students. Although acceptable reliability metrics were found for the components, the questionnaire received criticism for containing too many items [25]. This led to the revision of the instrument by Peter Gu in 2018. Gu shortened the questionnaire, down from 108 items to 62 items, and tried to cover two major psychological factors affecting vocabulary learning: Beliefs and Strategies [21].

In Gu's study [21], Vocabulary Learning Beliefs (VLB) were categorized into beliefs in *Word Use* and beliefs in *Word Memorization*. Word memorization beliefs examine learners' attitudes toward deliberate, intentional word learning while the Word Use category contains questions about the productive aspects of the word and incidental vocabulary acquisition through reading. 52 items of Gu's (2018) questionnaire were devoted to 7 umbrella Vocabulary Learning Strategies (VLS): *Meta-cognitive strategies, Inferencing, Using dictionary, Taking notes, Rehearsal, Encoding* and *Activation*. Some strategies like Meta-cognitive strategies, Taking notes, Rehearsal, and Encoding were further divided into other sub-categories that deal with very specific VLSs.

Despite being improved, Gu's Vocabulary Learning Questionnaire (VLQ) still has limitations that hinder its potential. One of the very first concerns was about the validation technique applied in Gu's study. In an attempt to provide validity evidence for the VLQ, Gu [21] employed exploratory factor analyses (EFA) and Cronbach's α as his main validation techniques. The 62-item VLQ and its multidimensionality have not been examined and confirmed by confirmatory factor analyses (CFA). The use of Cronbach's α as the single reliability metric has also received heavy criticism [26,27] due to the strict assumption of tau-equivalence. It is clear in Gu's (2018, pp. 336–338) that most of the β values suggested a severe violation of this assumption. These suggest that the construct validity of the instruments needs to be re-examined using stricter statistical techniques. The second constraint concerns the length of VLQ. In a recent study, Chou [22] criticized Gu's VLQ for being too long and time-consuming. Chou's study showed that the number of questionnaire items could be further reduced through PCA and CFA, allowing the VLQ to be more time-efficient. Reducing the time and effort needed to complete the questionnaire would increase its practicality, thus making the instrument more feasible for administration in offline classroom settings.

The third issue of the VLQ lies with the construct-irrelevant noise caused by language-related constructs (e.g. vocabulary and grammar knowledge). When improving the VLQ, Gu tried to minimize the lexical difficulty of the items by limiting the occurrence of mid- and low-frequency words as well as academic words, meta-language jargons were also limited and replaced by concrete examples where possible. The items were also made grammatically simple when only the use of simple tenses was allowed. However, we would argue that these simplifications would not be enough for a measurement instrument to be administered in an English as a Foreign Language (EFL) context [12, 28, 29]. As a learning belief and strategy questionnaire, the VLQ must be able to measure what it is designed to measure and be useable for learners of all levels of English proficiency. In all cases, the only method to completely exclude this language-related construct-irrelevant variance is to administer the instrument in the participants’ first language (L1). Otherwise, we would explicitly allow vocabulary and grammatical knowledge to interfere with our constructs.

As a result, this study was carried out to (1) develop a Vietnamese version of Gu's (2018) questionnaire, (2) to have its multidimensionality tested with both EFA and CFA, (3) and to see if there was a possibility to reduce the number of items further and to make the questionnaire more effort- and time-efficient for future use. In addition, while criticizing Gu for using α as the measure of internal consistency metrics, we also wish to compare the alphas found in this study to another highly-regarded metric of reliability, the omega, to see if our critics were really in evidence.

## 2 Methodology

### 2.1 Participants

A total of 722 students majoring in various subjects from a top-tier university in Vietnam participated in the study. The students’ ages ranged from 19 to 21. Of the 722 participants, 171 (23.7%) were males and 551 (76.3%) were females. As the study includes 722 observations and represents a subject-to-variable (STV) ratio of 11.6, it satisfies all the criteria concerning an appropriate sample size for factor analysis [[30], [31], [32], [33], [34]].

### 2.2 Instruments

A Vietnamese version of Gu's (2018) 62-item VLS questionnaire was used. The present study employed the likelihood-based, 7-point Likert scale used in the paper-based version of Gu's questionnaire, ranging from ‘extremely untrue of me’ to ‘extremely true of me’. The questionnaire items and options were translated into Vietnamese by the first author, who holds a Ph.D. degree in English Linguistics and were then carefully reviewed and revised by all the authors who are English Language Lecturers at a top-tier university in Vietnam. All the authors are native Vietnamese speakers and advanced English users who scored 7.5 or above in the International English Language Testing System (IELTS). The translated questionnaire was then piloted with 20 undergraduates from another university to see if the translated items were comprehensible. No difficulties in understanding and completing the questionnaire were reported.

In the present study, the items in the VLQ were tagged to reflect better the category of belief or strategy they belonged. This was done with the aim of making the article more readable as readers would have better ideas about which items or which categories of items were being referred to. The full list of 62 tagged items is presented in our Supplementary Files, Appendix 1, this was also the original of Gu's (2018) VLQ.

### 2.3 Data collection

A total of 1200 students were invited to complete the Vietnamese VLQ online through Google Form at their convenience. The studies involving human participants were reviewed and approved by University of Economics Ho Chi Minh City (UEH). A written informed consent form was sent to the participants before the questionnaire. The consent form introduced the research team, the study's relevance and objectives as well as how the confidentiality and anonymity of collected data were warranted. Students were also informed that they could withdraw from the study at any time and that their decision not to participate in the study would not affect either their academic results or their relationships with the researchers. Only students who electronically signed the consent form could proceed to complete the questionnaire. 722 students voluntarily answered and completed the questionnaire, representing a 60.16% response rate and a 722/62 = 11.6 Subject-to-Variable (STV) ratio.

### 2.4 Data analysis

Collected data on Google Form was first exported to Excel, and reversed coding was applied for items Meta2.2, Meta2.3 and Meta2.4. Data were then imported to Jamovi 2.3.13 for data analysis. Jamovi is a free statistical software package for social science research [35]. The present study employed Jamovi, incorporated with the SEMLj module [36] based on lavaan syntax [37] to conduct Exploratory Factor Analyses (EFA), Confirmatory Factor Analyses (CFA), and Covariance-based Structural Equation Modeling (CB-SEM).

Descriptive statistics were examined before factor analyses could be conducted. The mean values of the items ranged from 3.28 to 6.22 with standard deviations ranging from 0.965 to 1.56. The metrics of Skewness and Kurtosis coefficients were within the range of ±2 and ± 5, in the order given, suggesting a normal contribution of data [38,39].

EFA, CFA, and CB-SEM were performed to confirm the factors and models hypothesized in the Vietnamese VLQ. As the VLQ contains two distinct dimensions of vocabulary learning beliefs (10 items) and vocabulary learning strategies (52 items), separate factor analyses were conducted for each factor (Belief and Strategy). Once two structural equation models had been built for both Vocabulary Learning Belief (VLB) and Vocabulary Learning Strategies (VLS) in the second order, two exogenous variables (VLB and VLS) were correlated, which formed the final structural equation model.

As one of the study's purposes involved reducing the number of VLQ's items, for EFA, the items (10 for VLB and 52 for VLS) were subjected to principal component analyses (PCA) with Varimax rotation. Only components with an eigenvalue greater than 1 were taken into account. Items with factor loadings below 0.30 were excluded. Items that were cross-loaded at 0.30 and above were also deleted.

For CFA and CB-SEM, the following metrics with the criteria put forward by Kline [38,39] and Zhu, Raquel and Aryadoust [40] were used to evaluate model fit: The Chi-square test with χ^2^/df ratio lower than 3.0; the Goodness of Fit Index (GFI) greater than 0.9; the Comparative Fit Index (CFI) larger than 0.9; non-norm fit index or the Tucker-Lewis Index (TLI) from 0.9 and above. The values of the Root Mean Square Error of Approximation (RMSEA) and Standardized Root Mean Square Residual (SRMR) were expected to be lower than 0.05 and 0.08, respectively, for a good model fit.

Both Cronbach's α and McDonald's ω were used to estimate the components' reliability in the present study. The use of ω in addition to the traditional α was due to an easily violated assumption of α concerning equal tau coefficients. Hayes and Coutts (p.20) [26] said, “Cronbach's α is a special case of ω that requires a restrictive assumption that is unlikely to be met in many measurement situations. […] Thus, clinging to α because it is easier to calculate is an untenable position to take”. Over the past few years, many researchers have abandoned the old α and employed other superior replacements. Or, as McNeish (p.1) [27] put it in an affirmative tone: “Thanks coefficient alpha, we'll take it from here”.

## 3 Results

### 3.1 vocabulary learning beliefs

When EFA was first performed for the 10 items of VLB, the PCA revealed the presence of 2 components with eigenvalues greater than 1. The first component included 6 items (Belief1.1 – Belief1.6) and the second component consisted of 4 items (Belief2.1 – Belief2.4). All the items loaded with values larger than 0.5 and no cross-loadings were detected. The Kaiser-Meyer-Olkin (KMO) was 0.822 and Test of Sphericity achieved statistical significance results, generally suggesting suitability for structural detection. However, when CFA was conducted, the modification model suggested that 4 items should be excluded for high χ^2^ values. This resulted in the deletion of Belief1.1, Belief1.2, Belief1.4 and Belief2.2.

Table 1 shows the results of the second PCA on the remaining 6 items. The two components explained 62.6% of the variance. The KMO value in this round was 0.723, still larger than the recommended value of 0.6. Statistical significance was reached for the Test of Sphericity. The two metrics of reliability of the two latent constructs were above the recommended value of 0.65.

**Table 1:** Factor loadings and reliability metrics of vocabulary learning belief.

Items	Factor Loadings	
	1	2
Belief1.3		0.797
Belief1.5		0.755
Belief1.6		0.738
Belief2.1	0.812	
Belief2.3	0.822	
Belief2.4	0.769	
Cronbach's α	0.739	0.653
McDonald's ω	0.741	0.656

**Fig. 1:**
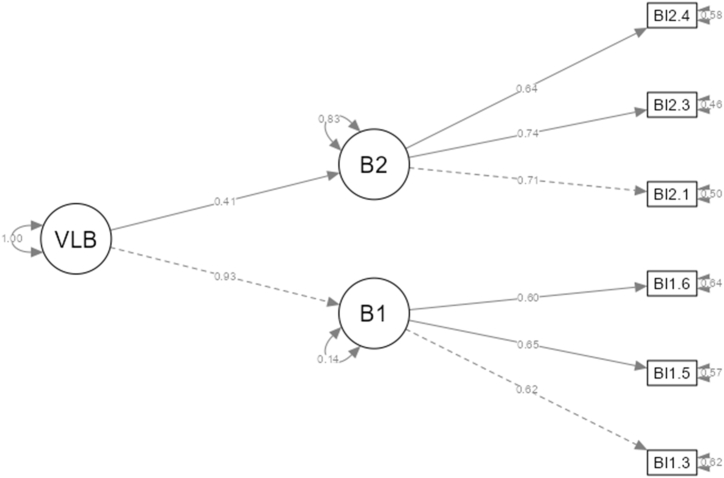
Vocabulary learning beliefs. *Note: VLB = Vocabulary Learning Belief; Bl1.3–2.4 = Belief1.3–2.4.

The 6 remaining items of VLB were once again examined with CFA. The results from the CFA for the first-order model suggested a near-perfect overall model fit: χ^2^/df = 11.7/8 = 1.46, RMSEA = 0.025, SRMR = 0.021, CFI = 0.996, TLI = 0.992, GFI = 1.000. Similarly, the standardized path for the second-order model showed a very good general model fit: χ^2^/df = 11.7/7 = 1.67, RMSEA = 0.030, SRMR = 0.021, CFI = 0.994, TLI = 0.988, GFI = 1.000. Fig. 1 demonstrates the second-order CFA model for VLB.

### 3.2 Vocabulary learning strategy

PCA was first performed for 52 items of the VLS. Results from the analyses suggested the exclusion of 25 items for low-factor loadings and cross-loading. Later CFA suggested that another 3 items be deleted for very low β values. The list of items deleted is illustrated in Supplementary Files, Appendix 1. The remaining 24 items were subject to the second round of PCA with Varimax rotation. Overall KMO test result was 0.918 and Bartlett's Test of Sphericity achieved statistically significant results. As a result, a 7-factor solution with 24 items was accepted, explaining 72.1% of the total variance. The metrics of α and ω suggested that all the components reached the acceptable range for reliability. The second-round PCA results are demonstrated in Table 2.

**Table 2:** Factor loadings and reliability metrics of vocabulary learning strategy.

Items	Factor loadings						
	1	2	3	4	5	6	7
Meta1.1							0.771
Meta1.2							0.788
Meta1.3							0.662
Infer1	0.753						
Infer 2	0.780						
Infer 3	0.761						
Infer 4	0.764						
Infer 5	0.718						
Dic5						0.754	
Dic6						0.808	
Dic7						0.754	
Note1.1					0.778		
Note1.2					0.801		
Note1.3					0.752		
Rehear3.1			0.867				
Rehear3.2			0.818				
Rehear3.3			0.834				
Encode3.1				0.776			
Encode3.2				0.777			
Encode3.3				0.778			
Activ1		0.763					
Activ2		0.776					
Activ3		0.786					
Activ4		0.718					
α	0.871	0.867	0.848	0.824	0.801	0.834	0.744
ω	0.871	0.870	0.852	0.824	0.801	0.837	0.749

**Fig. 2:**
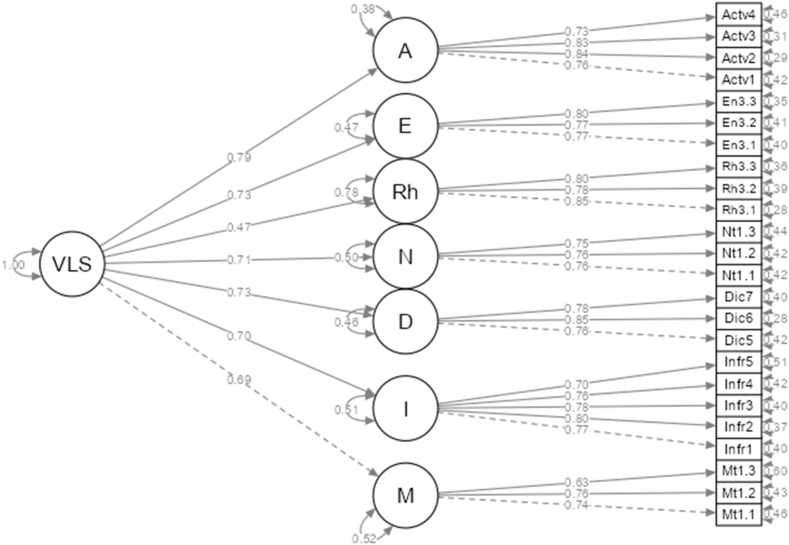
Vocabulary learning strategies. *Note: VLS = Vocabulary Learning Strategy; Actv1-4 = Active 1-4; En3.1–3.3 = Encode3.1–3.3; Rh3.1–3.3 = Rehear3.1–3.3; Nt1.1–1.3 = Note1.1–1.3; Infr1-5 = Infer1-5; Mt1.1–1.3 = Meta1.1–1.3.

The 24 items were again examined with a CFA. The standardized path coefficients for the first-order model suggested a healthy model fit: χ^2^/df = 457/231 = 1.98, RMSEA = 0.037, SRMR = 0.033, CFI = 0.973, TLI = 0.968, GFI = 0.992. Results from the second-order CFA model also showed an acceptable model fit: χ^2^/df = 589/245 = 2.40, RMSEA = 0.044, SRMR = 0.050, CFI = 0.960, TLI = 0.954, GFI = 0.990. Fig. 2 shows the second-model CFA model for VLS.

### 3.3 Vocabulary learning belief and strategy

**Fig. 3:**
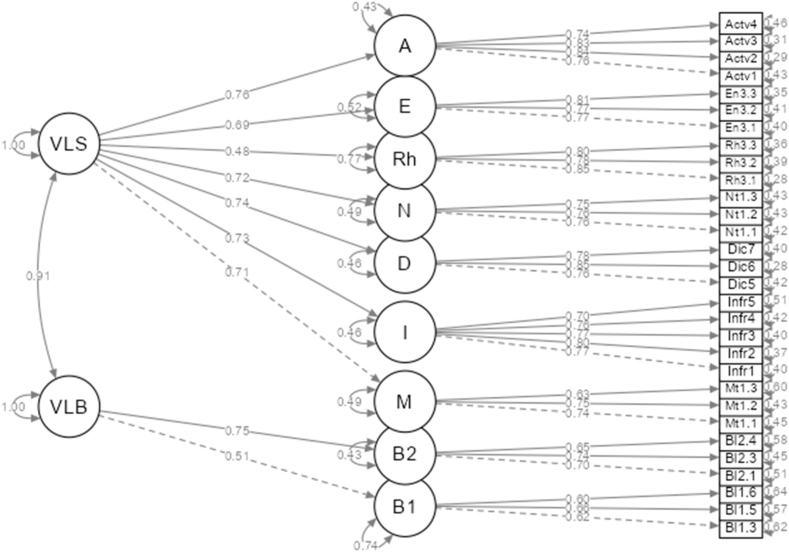
A hierarchical model of vocabulary learning strategy and vocabulary learning belief.

After good models for both VLS and VLB were established. The interest was then in the correlation between the two models. As a result, a structural equation model for both VLS and VLS was formed. The CFA returned a decent model fit: χ^2^/df = 957/395 = 2.42, RMSEA = 0.044, SRMR = 0.056, CFI = 0.943, TLI = 0.937, GFI = 0.987. Fig. 3 illustrates the CFA model. This second-order CFA model validates the hypothesized latent structure through which general indices of VLB and VLS could be obtained. The weights of the first-order factors, as reflected in the β values, implied that, except Rehearsal (Rh) (β = 0.476, R^2^ = 0.23), all vocabulary strategies were of similar importance. Word-use beliefs (B2) (β = 0.753, R^2^ = 0.57) were found to be more important to vocabulary learning compared to Word-memorization beliefs (B1) (β = 0.510, R^2^ = 0.26). Positive, moderate to strong correlations were spotted between the factors (Table 3). The two exogenous variables of VLB and VLS were also strongly correlated, highlighting the relationship between belief and strategy.

**Table 3:** Correlation matrix of the factors.

Factor	B1	B2	M	I	D	N	Rh	E	A
B1	–								
B2	0.266**	–							
M	0.268**	0.403**	–						
I	0.180**	0.518**	0.498**	–					
D	0.148**	0.447**	0.400**	0.544**	–				
N	0.311**	0.379**	0.364**	0.401**	0.432**	–			
Rh	0.419**	0.191**	0.339**	0.178**	0.204**	0.336**	–		
E	0.254**	0.270**	0.380**	0.360**	0.413**	0.433**	0.391**	–	
A	0.237**	0.355**	0.405**	0.459**	0.502**	0.465**	0.345**	0.535**	–

**p < 0.01.

## 4 Discussion

The purpose of the current study was to validate a Vietnamese version of the self-report questionnaire to assess English learners' beliefs and strategies in vocabulary learning. The research analyzed the factorial structure of vocabulary belief and strategy in isolation to see if these beliefs and strategies should be considered as individual, separated first-order factors or whether there's a possibility of merging them together into second-order factors. More importantly, the study offered insight into the correlation between the two models of VLS and VLB, providing evidence for the construct validity of the factor structure. Though the exclusion of certain items due to low factor loadings, cross-loading, and high χ^2^ values, the research introduced a new, smaller scale that greatly reduces teachers' and students' time and effort. The 30-item VLQ would only require half as much time prepared to complete compared to the original version. Second-order CFA models of both VLB and VLS showed very good model fits, generally offering cross-validation evidence for the first-order constructs and suggesting the possibility of obtaining general indices of VLBs and VLSs [41].

Compared with Gu's [21] 14 factors (62 items) extracted from EFA and Chou's [25] 9 factors (33 items) extracted from CFA, the present study's 9 factors with 30 items seem to be comparable (Supplementary Files, Appendix 1). In fact, except that Visual and Audio Encoding factors were excluded for cross-loading, and Self-initiation was omitted for low β values, the remaining factors between the present study and Chou [25] share a great similarity. It should be noted that, despite the deletion of certain first-order factors (Supplementary Files, Appendix 1), all the second-order VLS factors in Gu's study (Metacognitive strategies, Inferencing, Using Dictionary, Note-taking, Rehearsal, Encoding and Activation), which are the core of the questionnaire, remained intact.

In Chou's (2022) study, VLB and VLS were also examined separately, and factors of vocabulary beliefs were deleted for low-reliability values. In the present study, not-very-high reliability values were also recorded for the two Belief factors, especially Word Memorization beliefs (B1). However, we considered these reliability indices to be within the acceptable range [[42], [43], [44]] and deemed the two components retainable. Keeping the two components of VLS and VLB is significant to the Vietnamese VLQ as it would allow later studies to investigate the relationship between VLS, VLB, and different types of vocabulary knowledge.

The study also sheds light upon the two reliability metrics of α and ω. As clearly shown in Table 1, Table 2, near-identical similarities were found between the two indices of reliability for each factor. It is also worth noting that, in most of our cases, the essential tau-equivalence assumption of α was violated. The findings were consistent with many others in the field. Even Hayes and Coutts (p.20) [26] had to say:

However, anyone who adopts this recommendation will eventually discover something that we should be honest and open about. While the methodology literature has demonstrated that ω is clearly a superior and preferred measure of reliability relative to α, in our experience, the two measures typically produce similar estimates of reliability when applied to real data.

This suggested that Gu's use of α might not be problematic in the first place. These findings, together with other advantages of the α, suggest that α should not be completely replaced, but to be used in parallel with other metrics like ω or composite reliability. And maybe, at some points, we would have to cite Raykov and Marcoulides (p.1) [45], in a grateful tone: “Thanks Coefficient Alpha, We Still Need You!”

## 5 Conclusion

The present study sought to validate the multidimensional features of a translated and shortened version of Gu's (2018) VLQ, while maintaining the key factors of Vocabulary Learning Belief including word memorization and word use, and Vocabulary Learning Strategies such as selective attention, guessing, dictionary, note-taking, visual repetition, word form, and activation. Overall, the 9-factor, 30-item shows acceptable reliability and validity, offering a good instrument for measuring vocabulary learning beliefs and strategies of Vietnamese learners of English. Teachers could use data collected from the questionnaire to gain useful information about their students' beliefs and strategies for vocabulary learning.

Although results from the CFA confirmed the multidimensionality of the constructs, several limitations tempered the findings. The first weakness was that measures of VLB and VLS did not come with measures of vocabulary knowledge. As a result, this limitation restricted our understanding on the relationship between the three variables as well as how students’ vocabulary knowledge differ as a result of their VLB and VLS. Future studies on this are definitely encouraged. Secondly, despite a large sample size, all of the learners participated in the study were undergraduates. This constraint may restrain the generalizability of findings to other populations of learners (high school or secondary school students). Including learners from different backgrounds would not only further strengthen the validity of the questionnaire but can also shed light upon the degree to which VLB and VLS differ from different background groups.

Even with these limitations, the present study bears valuable implications. Theoretically, this study is the first to include both the construct of VLB and VLS in a structural equation model and offer deeper insights into the relationship between the two underlying components of Gu's (2018). From a practical perspective, the validation of the Vietnamese VLQ offers teachers and researchers in the context instruments for measuring English learners' VLB and VLS without the concerns about L2-related construct-irrelevant noises, generally supporting vocabulary teaching and learning research in the region.

## Funding

This research is funded by University of Economics Ho Chi Minh City, Vietnam (UEH).

## Author contribution statement

Nguyen Huynh Trang: Conceived and designed the experiments; Performed the experiments; Contributed reagents, materials, analysis tools or data; Wrote the paper.

Duy Vinh Truong: Conceived and designed the experiments; Contributed reagents, materials, analysis tools or data; Wrote the paper.

Hung Tan Ha: Conceived and designed the experiments; Analyzed and interpreted the data; Wrote the paper.

## Data availability statement

Data included in article/supplementary material/referenced in article.

### Ethics approval

The studies involving human participants were reviewed and approved by University of Economics Ho Chi Minh City (UEH).

## Consent to participate

The participants provided their written informed consent to participate in this study.

## Declaration of competing interest

The authors declare that they have no known competing financial interests or personal relationships that could have appeared to influence the work reported in this paper
