# Supplementary material for: Quantifying vocabulary learning belief and strategy - A validation study of the Vietnamese version of Gu's (2018) vocabulary learning questionnaire
Source: Heliyon. 2023 May 1;9(5):e16009. doi: 10.1016/j.heliyon.2023.e16009 (PMC10176061; doi:10.1016/j.heliyon.2023.e16009)
Supplement: Multimedia component 2 [file mmc2.docx]

Appendix 1

Gu’s (2018) Vocabulary Learning Questionnaire, with items tagged and deleted items highlighted

| Categories | Strategies | Tag | Items |
| --- | --- | --- | --- |
| Beliefs about vocabulary learning  (VLB) | Words should be memorized  (B1) | ~~Belief1.1~~ | ~~1. Once the English words of all my native language meanings have been remembered, English is learned.~~ |
|  |  | ~~Belief1.2~~ | ~~2. The best way to remember words is to memorize word lists or dictionaries.~~ |
|  |  | Belief1.3 | 3. The purpose of learning a word is to remember it. |
|  |  | ~~Belief1.4~~ | ~~4. A good memory is all you need to learn a foreign language well.~~ |
|  |  | Belief1.5 | 5. Repetition is the best way to remember words. |
|  |  | Belief1.6 | 6. You can only learn a large vocabulary by memorizing a lot of words. |
|  | Words should be learned through use  (B2) | Belief2.1 | 7. The meanings of a large amount of words can be picked up through reading. |
|  |  | ~~Belief2.2~~ | ~~8. Learners should pay attention to expressions (e.g., pick up) and collocations (e.g., heavy rain; strong wind) that go with a word.~~ |
|  |  | Belief2.3 | 9. Learners can learn vocabulary simply through reading a lot. |
|  |  | Belief2.4 | 10. The least a learner should know about a word is its spelling, pronunciation, meaning, and its basic usage. |
| Metacognitive strategies  (VLS) (M) | Selective attention | Meta1.1 | 11. I know whether a new word is important in understanding a passage. |
|  |  | Meta1.2 | 12. I know which words are important for me to learn. |
|  |  | Meta1.3 | 13. When I meet a new word or phrase, I know clearly whether I need to remember it. |
|  | ~~Self-initiation~~ | ~~Meta2.1~~ | ~~14. Besides textbooks, I look for other readings that fall under my interest.~~ |
|  |  | ~~Meta2.2~~ | ~~15. I wouldn’t learn what my English teacher doesn’t tell me to learn. (Reversed value)~~ |
|  |  | ~~Meta2.3~~ | ~~16. I only focus on things that are directly related to examinations. (Reversed value)~~ |
|  |  | ~~Meta2.4~~ | ~~17. I wouldn’t care much about vocabulary items that my teacher does not explain in class. (Reversed value)~~ |
| Inferencing  (VLS) (I) | Guessing strategies | Infer1 | 18. I make use of the logical development in the context (e.g., cause and effect) when guessing the meaning of a word. |
|  |  | Infer2 | 19. I use common sense and knowledge of the world when guessing the meaning of a word. |
|  |  | Infer3 | 20. I check my guessed meaning in the paragraph or whole text to see if it fits in. |
|  |  | Infer4 | 21. When I don’t know a new word in reading, I use my background knowledge of the topic to guess the meaning of the new word. |
|  |  | Infer5 | 22. I look for explanations in the reading text that support my guess about the meaning of a word. |
|  |  | ~~Infer6~~ | ~~23. I make use of the grammatical structure of a sentence when guessing the meaning of a new word.~~ |
|  |  | ~~Infer7~~ | ~~24. I make use of the part of speech of a new word when guessing its meaning.~~ |
| Using dictionary  (VLS) (D) | Dictionary strategies | ~~Dic1~~ | ~~25. When I see an unfamiliar word again and again, I look it up.~~ |
|  |  | ~~Dic2~~ | ~~26. When not knowing a word prevents me from understanding a whole sentence or even a whole paragraph, I look it up.~~ |
|  |  | ~~Dic3~~ | ~~27. I look up words that are important to the understanding of the sentence or paragraph in which it appears.~~ |
|  |  | ~~Dic4~~ | ~~28. I pay attention to the examples when I look up a word in a dictionary.~~ |
|  |  | Dic5 | 29. When I want to have some deeper knowledge about a word that I already know, I look it up. |
|  |  | Dic6 | 30. When I want to know more about the usage of a word that I know, I look it up. |
|  |  | Dic7 | 31. I check the dictionary when I want to find out the similarities and differences between the meanings of related words. |
| Taking notes  (VLS) (N) | Choosing which word to put into notebook | Note1.1 | 32. I make a note when I think the meaning of the word I’m looking up is commonly used. |
|  |  | Note1.2 | 33. I make a note when I think the word I’m looking up is related to my personal interest. |
|  |  | Note1.3 | 34. I make a note when I see a useful expression or phrase. |
|  | ~~Deciding what information goes into notes~~ | ~~Note2.1~~ | ~~35. I write down the English explanations of the word I look up.~~ |
|  |  | ~~Note2.2~~ | ~~36. I write down both the meaning in my native language and the English explanation of the word I look up.~~ |
|  |  | ~~Note2.3~~ | ~~37. I note down examples showing the usages of the word I look up.~~ |
| Rehearsal  (VLS) (Rh) | ~~Use of word lists~~ | ~~Rehear1.1~~ | ~~38. I go through my vocabulary list several times until I remember all the words on the list.~~ |
|  |  | ~~Rehear1.2~~ | ~~39. I make vocabulary cards and take them with me wherever I go.~~ |
|  |  | ~~Rehear1.3~~ | ~~40. I make regular reviews of new words I have memorized.~~ |
|  | ~~Oral repetition~~ | ~~Rehear2.1~~ | ~~41. When I try to remember a word, I say it aloud to myself.~~ |
|  |  | ~~Rehear2.2~~ | ~~42. When I try to remember a word, I repeat its pronunciation in my mind.~~ |
|  |  | ~~Rehear2.3~~ | ~~43. Repeating the sound of a new word to myself would be enough for me to remember the word.~~ |
|  | Visual repetition | Rehear3.1 | 44. When I try to remember a word, I write it again and again. |
|  |  | Rehear3.2 | 45. I memorize the spelling of a word letter by letter. |
|  |  | Rehear3.3 | 46. I write both the new words and their translation in my native language again and again in order to remember them. |
| Encoding  (VLS) (E) | ~~Visual encoding~~ | ~~Encode1.1~~ | ~~47. I act out some words in order to remember them better (e.g., jump).~~ |
|  |  | ~~Encode1.2~~ | ~~48. I create a picture in my mind to help me remember a new word.~~ |
|  |  | ~~Encode1.3~~ | ~~49. To help me remember a word, I try to “see” the spelling of the word in my mind.~~ |
|  | ~~Auditory encoding~~ | ~~Encode2.1~~ | ~~50. I put words that sound similar together in order to remember them.~~ |
|  |  | ~~Encode2.2~~ | ~~51. When words are spelled similarly, I remember them together.~~ |
|  |  | ~~Encode2.3~~ | ~~52. When I try to remember a new word, I link it to a sound-alike word that I know.~~ |
|  | Use of word-structure | Encode3.1 | 53. When I learn new words, I pay attention to prefixes, roots, and suffixes (e.g., inter-nation-al). |
|  |  | Encode3.2 | 54. I intentionally study how English words are formed in order to remember more words. |
|  |  | Encode3.3 | 55. I memorize the commonly used roots and prefixes. |
|  | ~~Contextual encoding~~ | ~~Encode4.1~~ | ~~56. When I try to remember a word, I also try to remember the sentence in which the word is used.~~ |
|  |  | ~~Encode4.2~~ | ~~57. I put words in set expressions or sentences in order to remember them.~~ |
|  |  | ~~Encode4.3~~ | ~~58. I remember a new word together with the context where the new word appears.~~ |
| Activation  (VLS) (A) | Activation | Activ1 | 59. I make up my own sentences using the words I just learned. |
|  |  | Activ2 | 60. I try to use the newly learned words as much as possible in speech and writing. |
|  |  | Activ3 | 61. I try to use newly learned words in real situations. |
|  |  | Activ4 | 62. I try to use newly learned words in imaginary situations in my mind. |

* Strikethroughs indicates deleted items.
