# Supplementary material for: Quantifying vocabulary learning belief and strategy - A validation study of the Vietnamese version of Gu's (2018) vocabulary learning questionnaire
Source: Heliyon. 2023 May 1;9(5):e16009. doi: 10.1016/j.heliyon.2023.e16009 (PMC10176061; doi:10.1016/j.heliyon.2023.e16009)
Supplement: Multimedia component 3 [file mmc3.docx]

Appendix 2

Vocabulary Learning Questionnaire

(Bảng khảo sát về "Chiến lược học từ vựng tiếng Anh")

The questionnaire belongs to one research project about vocabulary learning strategies. Three members are conducting the study.

(Đây là bảng khảo sát thuộc dự án nghiên cứu từ vựng của nhóm nghiên cứu gồm 3 giảng viên )XXX:

1. XXX (email: XXX)

2. XXX (email: XXX)

3. XXX (email: XXX)

The survey is part of research on vocabulary teaching and learning of Vietnamese students in general and XXX students in particular. The purpose of the study is to identify strategies students use when learning vocabulary. The study’s results will make an essential contribution to changing and improving the teaching and learning methods of vocabulary for Vietnamese students in particular and other countries learning English as a foreign language in general. And of course, this research could not have been completed without your contributions - students XXX.

(Bảng khảo sát là một phần trong nghiên cứu về việc dạy và học từ vựng của sinh viên Việt Nam nói chung và sinh viên XXX nói riêng. Mục đích của bảng khảo sát là nhằm xác định các chiến lược sinh viên sử dụng khi học từ vựng. Kết quả của nghiên cứu sẽ đóng góp một phần quan trọng vào việc thay đổi và cải tiến phương pháp dạy và học từ vựng cho sinh viên Việt Nam nói riêng và các nước học tiếng Anh như ngoại ngữ nói chung. Và dĩ nhiên, nghiên cứu này không thể được hoàn thành mà thiếu đi sự đóng góp của các bạn - sinh viên XXX.)

If you agree to participate in the study, you will attend as representatives of English learners in countries where English is learned as a foreign language and contribute to something of great significance - the development of English as a foreign language. of teaching and learning English not only for Vietnam but also for other countries.

(Nếu các bạn đồng ý tham gia nghiên cứu, các bạn sẽ tham gia với tư cách là đại diện cho người học tiếng Anh tại các nước nói tiếng Anh như ngoại ngữ, và đóng góp vào một điều mang ý nghĩa lớn - sự phát triển của việc dạy và học tiếng Anh không chỉ cho Việt Nam mà còn cho các nước khác.)

We appreciate your contributions as research participants. At the same time, we are also committed to complying with the ethical standards of researchers. Accordingly, we pledge to you the following:

(Chúng tôi trân trọng sự đóng góp của các bạn với tư cách là người tham gia nghiên cứu. Đồng thời chúng tôi cũng cam kết tuân thủ các quy chuẩn đạo đức của những nhà nghiên cứu. Theo đó, chúng tôi xin cam kết với các bạn những điều sau):

1. Your data will be absolutely confidential. Especially for information that can be used to identify you, no one other than members of the research team will have access. The survey data will be used for research purposes only. Therefore, please note that there is no right or wrong answer.

(1. Dữ liệu của các bạn sẽ được bảo mật một cách tuyệt đối. Đặc biệt là đối với những thông tin có thể dùng để định danh các bạn, thì ngoài thành viên của nhóm nghiên cứu sẽ không ai có quyền truy cập. Dữ liệu của bảng khảo sát sẽ chỉ được sử dụng vào mục đích nghiên cứu. Do đó, xin hãy lưu ý rằng sẽ không có câu trả lời nào được cho là đúng hoặc sai.)

2.You participate in the research on a voluntary basis and have the right to refuse to participate in the research. Even if you have agreed to participate in the research, you can email any member of the research team. and withdraw from the study at any time without giving a reason. Members of the research team will also not ask you the reasons.

(Các bạn tham gia nghiên cứu trên nguyên tắc tự nguyện và được quyền từ chối không tham gia vào nghiên cứu, Ngay cả khi các bạn đã đồng ý tham gia nghiên cứu, các bạn có thể email cho bất cứ thành viên nào của nhóm nghiên cứu và xin rút khỏi nghiên cứu vào bất cứ lúc nào mà không cần phải đưa lý do. Thành viên của nhóm nghiên cứu cũng sẽ không hỏi lý do từ các bạn.)

3. Whether or not you participate in the study will not affect the relationship between you and the research team members, nor will it impact the subjects you are taking with the team member. research (if any).

(3. Việc tham gia nghiên cứu hay không hoàn toàn không ảnh hưởng đến mối quan hệ giữa các bạn và các thành viên nhóm nghiên cứu, cũng sẽ không có bất kì tác động nào đến các môn học mà bạn đang tham dự với thành viên của nhóm nghiên cứu (nếu có).)

In case you have any questions or complaints about this study, you can contact the project manager, XXX via email: XXX

(Trong trường hợp các bạn có bất cứ thắc mắc hoặc khiếu nại về nghiên cứu này, các bạn có thể liên hệ chủ nhiệm đề tài, XXX qua email: XXX)

Thank you so much for taking part in the survey!

(Chân thành cảm ơn các bạn tham gia trả lời khảo sát!)

If you agree to participate in the survey, please enter your first and last name instead of your signature:

(Nếu bạn đồng ý tham gia khảo sát xin vui lòng ghi Họ và tên thay cho chữ ký)

Female: Male Female

(Giới tính: Nam Nữ)

Section 1_ Popular statements about learning vocabulary (Những nhận định phổ biến về việc học từ vựng)

INSTRUCTION (HƯỚNG DẪN)

Dear Students, please choose a number from 1 (strongly disagree) to 7 (strongly agree) corresponding to your level of agreement with each statement.

(Các bạn Sinh Viên vui lòng chọn một con số từ 1 (rất không đồng ý) đến 7 (rất đồng ý) tương ứng với mức độ đồng ý của các bạn với từng nhận định.)

| 1.1. Học từ vựng là phải học thuộc lòng (Beliefs about vocabulary learning) |
| --- |
| 1. Một từ tiếng Anh được coi là đã học thành công chỉ khi bạn nhớ được tất cả các nghĩa của từ ấy bằng ngôn ngữ mẹ đẻ.   ( Once the English words of all my native language meanings have been remembered, English is learned.)** |
| 1. Cách tốt nhất để ghi nhớ từ vựng là học thuộc từ theo một danh sách cố định hoặc từ điển.   (The best way to remember words is to memorize word lists or dictionaries.)** |
| 1. Mục đích của việc học từ vựng là nhớ được nó.   (The purpose of learning a word is to remember it.) |
| 1. Tất cả những gì bạn cần khi học ngôn ngữ là có một trí nhớ tốt.   (A good memory is all you need to learn a foreign language well.)** |
| 1. Lặp lại nhiều lần là cách tốt nhất để nhớ từ.   (Repetition is the best way to remember words.) |
| 1. Học thuộc nhiều từ là cách duy nhất giúp bạn sở hữu vốn từ vựng lớn cho bản thân.   (You can only learn a large vocabulary by memorizing a lot of words.) |

| 1.2. Học từ vựng là đang học cách sử dụng ngôn ngữ (Words should be learned through use) |
| --- |
| 1. Bạn sẽ học được nghĩa của nhiều từ thông qua việc đọc.   (The meanings of a large amount of words can be picked up through reading.) |
| 1. Bên cạnh học nghĩa của từ, người học cũng nên chú ý thêm về sắc thái, giá trị biểu cảm của từ ấy (ví dụ: pick up) và các cụm từ hay đi kèm với một từ (ví dụ: heavy rain, strong wind,...).**   (Learners should pay attention to expressions (e.g., pick up) and collocations (e.g., heavy rain; strong wind) that go with a word.) |
| 1. Đọc nhiều là cách đơn giản giúp chúng ta học được nhiều từ vựng.   (Learners can learn vocabulary simply through reading a lot.) |
| 1. Những khía cạnh cơ bản của một từ mà người học cần ghi nhớ là viết đúng chính tả, cách phát âm,ý nghĩa và cách sử dụng từ cơ bản.   (The least a learner should know about a word is its spelling, pronunciation, meaning, and its basic usage.) |

Section 2_Chiến thuật siêu nhận thức (Metacognitive strategies)

| 2.1. Sự sàng lọc (Selective attention) |
| --- |
| 1. Tôi biết từ mới nào quan trọng giúp tôi hiểu được bài đọc.   (I know whether a new word is important in understanding a passage.) |
| 1. Tôi nhận thức được những từ nào là quan trọng đối với tôi để học.   (I know which words are important for me to learn.) |
| 1. Khi tôi gặp một từ hoặc cụm từ mới, tôi biết rõ tôi có cần phải nhớ chúng hay không.   (When I meet a new word or phrase, I know clearly whether I need to remember it.) |
| 2.2. Sự tự giác (Self-initiation) |
| 1. Ngoài đọc sách giáo khoa, tôi còn tìm kiếm các bài đọc khác cùng chủ đề mà tôi đang quan tâm.   (Besides textbooks, I look for other readings that fall under my interest.)** |
| 1. Tôi sẽ không học những gì mà giáo viên tiếng Anh của tôi không bảo tôi học. (Đảo ngược giá trị)   (I wouldn’t learn what my English teacher doesn’t tell me to learn. (Reversed value)** |
| 1. Tôi chỉ tập trung vào những thứ có liên quan trực tiếp đến bài kiểm tra của tôi. (Đảo ngược giá trị)   (I only focus on things that are directly related to examinations.) (Reversed value)** |
| 1. Tôi sẽ không quan tâm đến những mục từ vựng mà giáo viên không giải thích trên lớp. (Đảo ngược giá trị)   (I wouldn’t care much about vocabulary items that my teacher does not explain in class. (Reversed value)** |

Section 3_Sự suy luận ( Inferencing)

Đoán nghĩa của từ (Guessing strategies)

| 1. Tôi tận dụng sự phát triển hợp lý của bối cảnh (ví dụ: nguyên nhân và kết quả) để đoán nghĩa của từ.   (I make use of the logical development in the context (e.g., cause and effect) when guessing the meaning of a word.) |
| --- |
| 1. Tôi sử dụng các kiến ​​thức thông thường và hiểu biết về thế giới để đoán nghĩa của từ.   (I use common sense and knowledge of the world when guessing the meaning of a word.) |
| 1. Tôi tự kiểm tra sự phán đoán nghĩa trong đoạn văn hoặc toàn bài đọc xem nó có phù hợp hay không.   (I check my guessed meaning in the paragraph or whole text to see if it fits in.) |
| 1. Khi tôi gặp từ mới trong bài đọc, tôi sẽ sử dụng kiến ​​thức nền tảng của mình và kiến thức liên quan đến chủ đề bài đọc để đoán nghĩa của từ.   (When I don’t know a new word in reading, I use my background knowledge of the topic to guess the meaning of the new word.) |
| 1. Tôi thường dựa vào lời giải thích trong bài đọc để giúp tôi suy đoán nghĩa của một từ mới.   (I look for explanations in the reading text that support my guess about the meaning of a word.) |
| 1. Tôi sử dụng cấu trúc ngữ pháp của một câu khi đoán nghĩa của từ mới.   (I make use of the grammatical structure of a sentence when guessing the meaning of a new word.)** |
| 1. Tôi dựa vào từ loại (danh từ, tính từ, động từ,..) để phán đoán nghĩa của từ.   (I make use of the part of speech of a new word when guessing its meaning.)** |

Section 4_Sử dụng từ điển (Using dictionary

Chiến lược tra từ điển (Dictionary strategies)

| 25. Khi tôi gặp một từ không quen thuộc được lặp đi lặp lại, tôi sẽ tra từ điển.  (When I see an unfamiliar word again and again, I look it up.)** |
| --- |
| 1. Tôi sẽ tra từ điển khi việc không biết nghĩa của từ khiến tôi không thể hiểu toàn bộ câu hoặc thậm chí cả đoạn văn.   (When not knowing a word prevents me from understanding a whole sentence or even a whole paragraph, I look it up.)** |
| 1. Tôi chỉ tra cứu những từ quan trọng có trong câu hoặc đoạn văn.   (I look up words that are important to the understanding of the sentence or paragraph in which it appears.)** |
| 1. Khi tra từ điển, tôi thường chú ý đến các ví dụ đi kèm.   ~~(~~I pay attention to the examples when I look up a word in a dictionary.)** |
| 1. Tôi sẽ tra từ điển khi tôi muốn hiểu sâu hơn về nghĩa của một từ mà tôi đã biết.   (When I want to have some deeper knowledge about a word that I already know, I look it up.) |
| 1. Tôi sẽ tra từ điển khi tôi muốn biết thêm về cách sử dụng của một từ mà tôi đã biết.   (When I want to know more about the usage of a word that I know, I look it up.) |
| 1. Tôi sẽ tra từ điển khi tôi muốn tìm hiểu thêm về sự giống và khác nhau về nghĩa của những từ liên quan.   (I check the dictionary when I want to find out the similarities and differences between the meanings of related words.) |

Section 5_Ghi chú (Taking notes)

| 5.1. Lựa chọn những từ để ghi vào sổ tay  (Choosing which word to put into notebook) |
| --- |
| 1. Tôi sẽ ghi chú lại khi tôi nghĩ rằng nghĩa của từ tôi đang tra cứu được sử dụng phổ biến.   (I make a note when I think the meaning of the word I’m looking up is commonly used.) |
| 1. Tôi sẽ ghi chú lại khi tôi cảm thấy từ tôi đang tra cứu có liên quan đến sở thích cá nhân của tôi.   (I make a note when I think the word I’m looking up is related to my personal interest.) |
| 1. Tôi sẽ ghi chú lại khi tôi thấy một cụm từ hay một đoạn văn nào hữu ích .   (I make a note when I see a useful expression or phrase.) |
| 5.2. Xác định những loại thông tin nào sẽ được ghi lại  (I write down the English explanations of the word I look up.) |
| 1. Tôi ghi lại cách giải nghĩa của từ mà tôi tra từ điển chỉ bằng tiếng Anh.   (I write down both the meaning in my native language and the English explanation of the word I look up.)** |
| 1. Tôi ghi lại cách giải thích của từ mà tôi tra từ điển bằng tiếng Anh và tiếng Việt.   (I write down both the meaning in my native language and the English explanation of the word I look up.)** |
| 1. Tôi có ghi lại các ví dụ cho thấy cách sử dụng của từ mà tôi đang tra cứu.   (I note down examples showing the usages of the word I look up)** |

Section 6_Ôn tập Rehearsal

| 6.1. Sử dụng các danh sách từ Use of word lists |
| --- |
| 1. Tôi xem qua các danh sách từ vựng của mình nhiều lần cho đến khi tôi nhớ được tất cả các từ trong danh sách ấy.   (I go through my vocabulary list several times until I remember all the words on the list.)** |
| 1. Tôi làm thẻ từ vựng và mang chúng theo mọi lúc mọi nơi.   (I make vocabulary cards and take them with me wherever I go.)** |
| 1. Tôi thường xuyên ôn lại những từ tôi đã học.   (I make regular reviews of new words I have memorized)** |
| 6.2. Lặp lại bằng âm thanh (Oral repetition) |
| 41. Khi tôi cố gắng nhớ một từ, tôi sẽ nói to từ đó lên. (When I try to remember a word, I say it aloud to myself.) |
| 42.Khi tôi cố gắng nhớ một từ, tôi sẽ lặp lại cách phát âm của từ đó trong đầu.  (When I try to remember a word, I repeat its pronunciation in my mind.)** |
| 43. Tôi cảm thấy việc lặp lại âm thanh của một từ mới là đủ để giúp tôi ghi nhớ từ đó.  (Repeating the sound of a new word to myself would be enough for me to remember the word.)** |
| 6.3. Lặp lại bằng hình ảnh  (Visual repetition) |
| 44. Tôi viết lại từ đó nhiều lần để ghi nhớ từ ấy.  (When I try to remember a word, I write it again and again.)*** |
| 45. Tôi học thuộc cách viết của từng từ.  (I memorize the spelling of a word letter by letter.) |
| 46. Tôi viết đi viết lại các từ mới và bản dịch của chúng bằng tiếng Việt để ghi nhớ chúng .  (I write both the new words and their translation in my native language again and again in order to remember them.) |

Section 7_Mã hóa (Encoding)

| 7.1. Mã hóa trực quan  Visual encoding |
| --- |
| 47. Để ghi nhớ từ vựng tốt hơn, tôi dùng hành động hoặc ngôn ngữ cơ thể để diễn tả (ví dụ: jump: nhảy).  (I act out some words in order to remember them better (e.g., jump). |
| 48. Tôi tự tạo ra một hình ảnh trong tâm trí để giúp tôi nhớ một từ mới.  (I create a picture in my mind to help me remember a new word.)** |
| 49. Để giúp tôi nhớ một từ, tôi cố gắng “xem” cách viết của từ đó trong tâm trí mình.  (To help me remember a word, I try to “see” the spelling of the word in my mind.)** |
|  |
| 7.2. Mã hóa âm thanh (Auditory encoding) |
| 1. Tôi nhóm những từ có cách phát âm gần giống nhau để ghi nhớ chúng tốt hơn.   (I put words that sound similar together in order to remember them.)** |
| 51. Khi các từ có cách viết gần giống nhau, tôi sẽ nhớ chúng cùng nhau.  When words are spelled similarly, I remember them together.** |
| 52. Khi tôi cố gắng nhớ một từ mới, tôi liên kết nó với một từ có cách phát âm tương tự mà tôi đã biết.  When I try to remember a new word, I link it to a sound-alike word that I know.** |
| 7.3. Mã hóa cấu tạo của từ Use of word-structure |
| 53. Khi tôi học từ mới, tôi chú ý đến tiền tố, hậu tố và gốc của từ đó (ví dụ: inter-nation-al).  When I learn new words, I pay attention to prefixes, roots, and suffixes (e.g., inter-nation-al). |
| 534 Tôi tự nghiên cứu cách mà các từ tiếng Anh được hình thành để sau này ghi nhớ nhiều từ hơn.  I intentionally study how English words are formed in order to remember more words. |
| 55. Tôi học thuộc các gốc từ và tiền tố thông dụng .  I memorize the commonly used roots and prefixes. |
| 7.4. Mã hóa ngữ cảnh Contextual encoding |
| 56. Khi tôi muốn nhớ một từ hoặc cụm từ mới, tôi sẽ cố gắng nhớ luôn cả câu mà từ đó được sử dụng.  When I try to remember a word, I also try to remember the sentence in which the word is used.** |
| 57. Tôi cảm thấy học từ mới thông qua các cụm từ cố định hoặc các câu hoàn chỉnh rất hiệu quả.  I put words in set expressions or sentences in order to remember them.** |
| 58. Tôi nhớ một từ cùng với ngữ cảnh mà nó xuất hiện.  I remember a new word together with the context where the new word appears.** |

Section 8_Kích hoạt/Vận dụng (Activation)

| 59. Tôi tự đặt câu bằng những từ vừa học được.  I make up my own sentences using the words I just learned. |
| --- |
| 60. Tôi cố gắng sử dụng những từ mới học được một cách nhiều nhất có thể trong bài nói và viết.  I try to use the newly learned words as much as possible in speech and writing. |
| 61. Tôi cố gắng sử dụng ngay những từ mới học được vào các tình huống thực tế.  I try to use newly learned words in real situations. |
| 62. Tôi cố gắng sử dụng những từ mới học được trong những tình huống tưởng tượng trong đầu.  I try to use newly learned words in imaginary situations in my mind. |

** indicates deleted items.

Thank you for taking the time to complete the survey!

Wishing you all good health and good study!

(Cám ơn các bạn đã dành thời gian hoàn thành bài khảo sát!

Chúc các bạn thật nhiều sức khỏe và học tập tốt!)
